# Efficacy of the Geriatric Trauma Outcome Score (GTOS) in Predicting Mortality in Trauma Patients: A Retrospective Cross-Sectional Study

**DOI:** 10.3390/diagnostics14232735

**Published:** 2024-12-05

**Authors:** Ching-Ya Huang, Shao-Chun Wu, Tsan-Shiun Lin, Pao-Jen Kuo, Johnson Chia-Shen Yang, Shiun-Yuan Hsu, Ching-Hua Hsieh

**Affiliations:** 1 Department of Plastic Surgery, Kaohsiung Chang Gung Memorial Hospital and Chang Gung University College of Medicine, Kaohsiung 83301, Taiwan; b101106030@tmu.edu.tw (C.-Y.H.); tslin@cgmh.org.tw (T.-S.L.); bow110470@gmail.com (P.-J.K.); prs581126@gmail.com (J.C.-S.Y.); 2 Department of Anesthesiology, Kaohsiung Chang Gung Memorial Hospital and Chang Gung University College of Medicine, Kaohsiung 83301, Taiwan; shaochunwu@gmail.com; 3 Department of Trauma Surgery, Kaohsiung Chang Gung Memorial Hospital and Chang Gung University College of Medicine, Kaohsiung 83301, Taiwan; ah.lucy@hotmail.com

**Keywords:** trauma, mortality, prognosis, Injury Severity Score (ISS), Geriatric Trauma Outcome Score (GTOS)

## Abstract

Background: Trauma has a profound impact on mortality as well as short- and long-term health outcomes. For trauma patients to receive medical care in a timely manner, early identification and risk assessment are essential. The Geriatric Trauma Outcome Score (GTOS), which was created by combining age, the Injury Severity Score (ISS), and the requirement for packed red blood cell transfusion, has proven to be a valuable prognostic tool for elderly trauma patients, though its applicability to general trauma patients is still understudied. Methods: This retrospective study analyzed data from the Trauma Registry System at a Level I trauma center in southern Taiwan, covering the period from 1 January 2009 to 31 December 2021. This study included 40,068 trauma patients aged 20 years and older. Statistical analyses included chi-square tests, ANOVA, Mann–Whitney U tests, and multivariate analyses to identify independent risk factors for mortality. The predictive performance of the GTOS was assessed using the area under the curve (AUC) of the receiver operating characteristic curve. Results: The final study population included 40,068 patients, with 818 deaths and 39,250 survivors. Deceased patients had higher GTOS scores (mean 132.8 vs. 76.1, *p* < 0.001) and required more blood transfusions (mean 4.0 vs. 0.3 units, *p* < 0.001) compared to survivors. The optimal GTOS cut-off value for predicting mortality was 104.5, with a sensitivity of 82.6% and a specificity of 84.3% (AUC = 0.917). A high GTOS score was associated with increased mortality (9.6 vs. 0.4%, *p* < 0.001) compared with a low GTOS score, even after adjusting for confounding factors (adjusted mortality rate of 2.86, *p* < 0.001), and a longer hospital stay (14.0 vs. 7.7 days, *p* < 0.001). Conclusions: The GTOS is a valuable prognostic tool for predicting mortality in trauma patients, providing a simple and rapid assessment method. Its high predictive accuracy supports its use in broader trauma patient populations beyond the elderly. Further studies are recommended to refine and validate the GTOS in diverse trauma settings to enhance its clinical utility.

## 1. Introduction

Trauma has a profound impact on mortality as well as short- and long-term health outcomes. A study of 124,421 trauma patients in Washington State found a 16% mortality rate within three years post-injury compared to 5.9% in the general population [1]. Prognostic predictions are vital in managing trauma patients by identifying and categorizing high-risk individuals and optimizing treatment to improve outcomes [2,3,4,5,6]. However, these systems often demand extensive data or specific baseline characteristics, which can be challenging to gather easily or quickly.

Effective screening, triage, and early risk stratification in trauma patients are paramount for ensuring timely and appropriate medical interventions, which can significantly enhance patient outcomes. Numerous studies have underscored the importance of early identification and risk stratification in trauma care. For instance, Tran et al. [7] highlighted the need for objective measures to identify patients with traumatic hemorrhage who may require massive transfusions or surgical interventions within the first hour of resuscitation. Similarly, Rainer et al. [8] developed a prediction rule to stratify the risk of massive transfusion needs based on the conscious level and physiological variables of heart rate and systolic blood pressure. There is a clear need for prognostic models that are simple and quick to calculate, requiring minimal data input and providing quick and actionable results. This need is underscored by the finding that many trauma deaths occur shortly after hospital admission, making rapid assessment and intervention critical [9].

In this landscape, the Geriatric Trauma Outcome Score (GTOS) emerges as a valuable prognostic measure specifically developed for elderly trauma patients [10,11,12,13,14,15,16,17]. This score incorporates age, the Injury Severity Score (ISS), and the necessity for packed red blood cell (PRBC) transfusion within 24 h after admission to offer a simple but effective predictor of in-hospital mortality. The formula is age + ISS × 2.5 + 22 (if any PRBCs were transfused within 24 h of admission). A study on 1080 geriatric trauma patients found that excluding those with care restrictions improved GTOS performance, with an area under the curve (AUC) of 0.88 [10]. Another validation study across multiple trauma centers confirmed the model’s accuracy, showing similar predictive metrics with an AUC of 0.86 [15]. Comparative studies revealed that the GTOS performs as well as or better than traditional scoring systems like the Trauma and Injury Severity Score (TRISS), with the advantage of using fewer variables and simpler calculations [18]. It has been demonstrated that the GTOS accurately predicts geriatric trauma patients [10,11,14,19,20,21,22,23,24,25] or patients with particular injuries like traumatic brain injury [26], but its application to general trauma patients is less explored. As a result, the purpose of this study is to assess the usefulness of the GTOS in expanding its field of applicability as a predictive tool for predicting mortality risk among adult trauma patients.

## 2. Methods

### 2.1. Patient Enrollment and Study Design

This study was approved by the Institutional Review Board (IRB) of Chang Gung Memorial Hospital under approval number 202400775B0. This study examined registered medical data from 1 January 2009 to 31 December 2021 from the Trauma Registry System in a Level I trauma center in southern Taiwan. The registered data in the Trauma database were input in a prospective way by two trained, accredited, full-time nurses and supervised by the corresponding attending. This study included all patients aged equal to or over twenty who had trauma. The exclusion criteria included patients with burns, hangings, drowning, and those with unavailable laboratory data. The research method involved detailed recording of all retrieved cases’ sex, age, blood transfusion history, GTOS, pre-existing comorbidities, Glasgow Coma Scale (GCS), Abbreviated Injury Scale (AIS), ISS, in-hospital mortality, and hospital stay. The AIS is the basis of the ISS, a standard for determining the severity of injuries [27,28,29,30,31]. Based on the degree of the injury, the scale scores each of the six anatomical regions (head/neck, face, thorium, abdomen, extremities, and external) from 1 to 6. Every region has a highest AIS score, and the ISS is determined by adding the squares of the three highest scores. The ISS ranges from 1 to 75, with higher numbers denoting more severe injuries [32].

The GTOS is calculated by adding the age to the product of the ISS multiplied by 2.5 and subsequently adding twenty-two if any packed red blood cells were administered within 24 h of arrival. To comprehensively review the literature on the GTOS, we conducted a systematic search of the PubMed, Embase, and Scopus databases. We used a combination of keywords and MeSH terms such as “GTOS”, “trauma mortality”, and “geriatric trauma”, applying Boolean operators to refine the search. The inclusion criteria focused on studies validating the GTOS, comparing it with other mortality prediction models, and involving trauma patients. Exclusion criteria included non-English publications, case reports, and conference abstracts. This process ensured a thorough and focused review of the most pertinent literature related to the GTOS.

### 2.2. Statistical Analysis

The chi-square test compared the proportions of categorical variables between deceased and surviving patients, calculating odds ratios (ORs) and 95% confidence intervals (CIs). Levene’s test ensured homogeneity of variances, followed by ANOVA to assess differences in continuous variables and the Mann–Whitney U test for non-normally distributed continuous variables (presented as median and interquartile range [IQR]). Univariate and multivariate analyses identified independent risk factors for mortality. The area under the receiver operating characteristic curve (AUC of ROC) determined the predictive performance and optimal cut-off value of the GTOS and identified independent risk factors using the Youden index [33,34]. An AUC value close to one denotes excellent predictive power, while an AUC value of 0.5 suggests no predictive ability. Adjusted odds ratios (AORs) were then computed for variables such as sex, age, comorbidities, and the ISS. The selection of these variables was based on their baseline characteristics and established influence on mortality in patients with trauma [22,35,36,37]. This study employed a propensity score-matched cohort analysis to compare outcomes between two groups while minimizing potential confounding factors. The propensity score matching was conducted using Numerical Computation for Scientific Software (NCSS) ver. 2022, employing a 1:1 greedy matching algorithm and the nearest neighbor caliper matching method with a caliper width equivalent to 0.2 standard deviations of the logit of the propensity score. This method facilitates the formation of equitable groups with comparable baseline characteristics such as age, sex, underlying health conditions, and their level of consciousness. This minimizes the possibility of bias in the assessment of treatment outcomes. Statistical analyses were performed with IBM SPSS Statistics, Version 23, with a significance threshold of *p* < 0.05.

## 3. Results

### 3.1. Enrollment of the Patients

**Figure 1:**
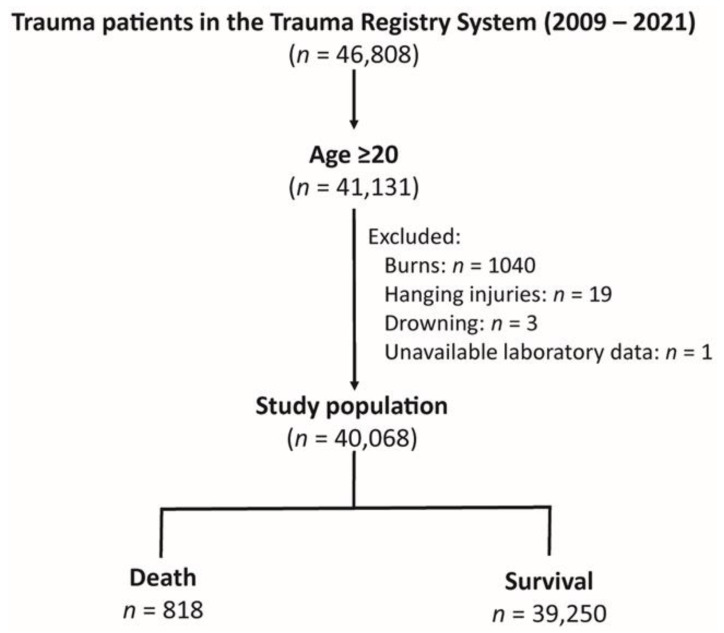
Enrollment process of the adult trauma patients into the study cohort.

The Trauma Registry System comprised 46,808 trauma patients from 2009 to 2021 for the research cohort (Figure 1). Patients twenty years of age or over made up 41,131 of the adult patients. The analysis was conducted on a final research population of 40,068 patients, after removing patients with burns (*n* = 1040), hanging injuries (*n* = 19), drowning (*n* = 3), and unavailable laboratory data (*n* = 1). In this group of patients, 39,250 people survived, while 818 people died.

### 3.2. Demographic and Clinical Characteristics of Patients Stratified by Outcomes

Table 1 presents the demographic characteristics and clinical variables for the study population. The patients who died were more likely to be male (66.6% vs. 54.1%, *p* < 0.001) and older (mean age 63.4 vs. 54.0 years, *p* < 0.001). They required more blood transfusions within the first 24 h (mean 4.0 vs. 0.3 units, *p* < 0.001) and had higher GTOS scores (mean 132.8 vs. 76.1, *p* < 0.001). The prevalence of comorbidities, including hypertension (HTN), coronary artery disease (CAD), and end-stage renal disease (ESRD), was higher among the deceased patients. The deceased patients had significantly lower median GCS scores (median 6 vs. 15, *p* < 0.001), with a higher percentage having scores in the 3–8 range (59.5% vs. 2.7%, *p* < 0.001). They also had higher rates of significant injuries (AIS ≥ 2) across various regions (head/neck, face, thorax, and abdomen) when compared to the survivors. A higher ISS was noted (median 25 vs. 8, *p* < 0.001) in those deceased patients, with a greater proportion having an ISS ≥ 25 (61.6% vs. 3.3%, *p* < 0.001). The length of hospital stay was longer for the deceased patients compared to the survivors (mean 11.0 vs. 8.7 days, *p* < 0.001). This may indicate that more severe conditions necessitate longer hospitalization, albeit the causality of this link is unclear.

### 3.3. Univariate and Multivariate Analysis of Factors Associated with Mortality

The univariate analysis in Table 2 showed that there was a significant correlation between increased mortality and male sex (OR 1.70, *p* < 0.001), older age (OR 1.03, *p* < 0.001), PRBC transfusion within 24 h (OR 1.25, *p* < 0.001), higher GTOS scores (OR 1.76, *p* < 0.001), and the presence of comorbidities like CAD (OR 2.86, *p* < 0.001), ESRD (OR 4.69, *p* < 0.001), HTN (OR 1.57, *p* < 0.001), and ESRD (OR 1.69, *p* < 0.001). An elevated ISS (OR 1.18, *p* < 0.001) was also associated with a higher death rate.

The GTOS score was identified as an independent risk factor associated with the mortality outcome (OR 1.37, 95%CI 1.32–1.51; *p* < 0.001). In addition, male (OR 1.62, *p* < 0.001), PRBC transfusion within 24 h (OR 1.06, *p* < 0.001), CAD (OR 1.38, *p* = 0.022) or ESRD (OR 4.47, *p* < 0.001), and a higher ISS (OR 1.09, *p* < 0.001) were found to be significantly linked with higher mortality in the multivariate analysis.

### 3.4. The Optimal Cut-Off Value of Independent Risk FACTORS in Predicting Mortality

**Figure 2:**
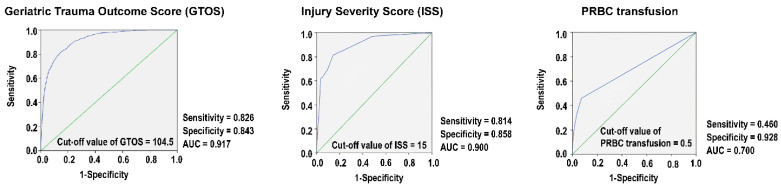
Performance characteristics of the Geriatric Trauma Outcome Score (GTOS), Injury Severity Score (ISS), and amount of packed red blood cell (PRBC) transfusion within the first 24 h for mortality prediction (blue line). The green line indicates an AUC = 0.5.

The ROC curve analysis for the GTOS shows that 104.5 is the best cut-off value for predicting mortality (Figure 2). At this cut-off, the sensitivity and specificity are 0.826 and 0.843, respectively. The AUC of 0.917 suggests that the GTOS is a good predictor of mortality risk for these trauma patients. Furthermore, the ISS exhibits an AUC of 0.900, a sensitivity of 0.814, and a specificity of 0.858 at a cut-off of 15. The amount of PRBC transfusion within 24 h shows an AUC of 0.700, a sensitivity of 0.460, and a specificity of 0.928 at the 0.5 cut-off value. The ISS, but not the PRBC transfusion, also presents as a favorable predictor of mortality risk in these trauma patients.

### 3.5. Comparative Demographics and Outcomes Based on Grouping by the GTOS

Table 3 compares the demographics and clinical outcomes of patients with high (≥104.5) versus low (<104.5) GTOS scores. The patients with a high GTOS were significantly older (mean age 72.1 vs. 50.4 years, *p* < 0.001), required more blood transfusions within the first 24 h (mean 1.64 vs. 0.1 units, *p* < 0.001), and had a higher prevalence of investigated comorbidities. The patients in the high GTOS group had lower GCS scores, with a higher percentage having scores in the range of 3–8 (14.7% vs. 1.5%, *p* < 0.001). They also had higher rates of significant injuries (AIS ≥ 2) across various regions: head/neck (77.3% vs. 17.1%, *p* < 0.001), face (11.6% vs. 9.0%, *p* = 0.010), thorax (24.6% vs. 10.7%, *p* < 0.001), and abdomen (11.2% vs. 5.3%, *p* < 0.001). Additionally, they had a higher ISS (median 16 vs. 4, *p* < 0.001), with a greater proportion having an ISS ≥25 (23.6% vs. 0.4%, *p* < 0.001). Mortality was significantly higher in the patients in the high GTOS group (9.6% vs. 0.4%, *p* < 0.001), and they had a significantly higher adjusted mortality rate (AOR 2.86, 95%CI: 2.20–3.73; *p* < 0.001) after adjusting for sex, age, comorbidities, and GCS. The patients with high GTOS scores also experienced longer hospital stays (mean 14.0 vs. 7.7 days, *p* < 0.001), which may indicate that these patients had more associated severe conditions requiring longer hospitalization, though the causality of this relationship is not clearly established.

### 3.6. Propensity Score-Matched Analysis of High and Low GTOS Score Groups

A comparison between patients with high (≥104.5) and low (<104.5) GTOS scores was performed using the propensity score-matched analysis (Table 4), with 3515 patients in each group. After matching, there were no appreciable variations in comorbidities, age, or sex across the groups. After attenuating the differences in baseline characteristics, the patients with a high GTOS score still had significantly higher mortality rates (6.7% vs. 2.8%, OR 2.50, 95%CI: 1.96–3.18; *p* < 0.001) and longer hospital stays (mean 14.8 vs. 9.6 days, *p* < 0.001) than those patients with a low GTOS score.

## 4. Discussion

Most studies of the GTOS focus on geriatric trauma patients aged 65 years and older, where the GTOS has been used for predicting disease prognosis and assessing risk, with AUCs ranging from 0.674 to 0.87 [10,11,13,15,20]. Additionally, studies have compared the GTOS to other commonly used prognostic tools, often demonstrating its superior performance. Madni et al. [18] further validated the GTOS’s advantage, citing its simplicity and fewer required variables compared to the TRISS. Studies by Zhao et al. [13] and Ravindranath et al. [11] found the GTOS to be more effective than age and the ISS alone, while Park et al. [20] demonstrated its higher predictive accuracy compared to the TRISS in Korean geriatric trauma patients, particularly those with a higher ISS. Similarly, these studies mostly focused on elderly patients and did not compare other prognostic tools to broader trauma patient groups. In this study, we identified a high AUC of 0.917 for the GTOS when applied to trauma patients. Historically, the GTOS was designed for elderly trauma populations (aged ≥65 years); however, our results demonstrate its robust performance in predicting in-hospital mortality for patients aged 20 years and older. Furthermore, the GTOS score is recognized as an independent risk factor that is associated with the mortality outcome. Patients who have a high GTOS score have a higher adjusted likelihood of mortality than those who have a low GTOS score, even in the propensity score-matched study cohort. This supports the broader applicability of the GTOS as a universal risk stratification tool in trauma care. However, since the GTOS relies on data such as the ISS and transfusion requirements within the first 24 h, its real-time utility in clinical decision-making during the early phases of trauma care is limited. Additionally, while many of the associations highlighted—such as the impact of sex, a higher ISS, the need for transfusions, and age on mortality—are well established in the trauma literature, our analysis aimed to confirm that the GTOS effectively integrates these variables into a composite score, aligning with the existing clinical knowledge and validating its predictive accuracy across a broad trauma cohort. Instead, the GTOS proves valuable for risk stratification among trauma patients who survive the initial resuscitation phase. In clinical practice, it aids in resource allocation, facilitates discussions about prognosis with patients and families, and supports prioritization in acute care settings. By providing a standardized and reliable method for predicting outcomes across diverse age groups, the GTOS complements existing prognostic tools while maintaining simplicity and ease of use.

An interesting and somewhat unexpected finding from our analysis is that age does not appear to play a significant role in the predictive power of the GTOS, despite being one of its core components. The GTOS is calculated based on age, the ISS, and the need for RBC transfusions. However, our multivariate analysis revealed that while the GTOS and ISS were significantly associated with increased mortality, age alone was not a strong predictor of outcomes. This suggests that the ISS and RBC transfusions may be more important determinants of mortality than age, which supports the broader application of the GTOS beyond the elderly population for which it was originally developed. These findings also highlight a critical limitation of using age as a surrogate for patient vulnerability. While age is a straightforward measure, it may not fully capture the complexity of factors that contribute to mortality, particularly in trauma patients. Frailty, a condition characterized by reduced physiological reserve and increased vulnerability to stressors, may provide a more nuanced and accurate assessment of a patient’s risk [38,39]. Unlike age, frailty encompasses multiple dimensions, including physical, cognitive, and social factors, which together offer a more comprehensive understanding of a patient’s overall health status [39,40]. Given the limitations of age as a predictor, it may be beneficial to reconsider the weight of age in the GTOS calculation. Incorporating frailty assessments into the GTOS may enhance its predictive accuracy, offering a more holistic approach to risk stratification.

It is worth mentioning that Barea-Mendoza et al. [25] discovered that the TRISS was marginally more effective in predicting survival among elderly patients. Furthermore, the GTOS demonstrated lower accuracy compared to two prognostic scores, the Acute Physiology and Chronic Health Evaluation (APACHE) III and Australian and New Zealand Risk of Death (ANZROD), in forecasting intensive care unit (ICU) mortality [11]. This finding underscores the need for further refinements to the GTOS to enhance its utility in predicting ICU outcomes specifically. Importantly, the role of ICU care in reducing mortality cannot be overstated, particularly in the context of trauma and emergency settings. The underutilization of ICU resources has been associated with significantly higher mortality rates, as demonstrated by Lasithiotakis et al. [41] in the Hellenic Emergency Laparotomy Study. Their study revealed that the lower rate of ICU admissions in Greek hospitals contributed to nearly double the postoperative mortality rate compared to British counterparts, highlighting the critical impact of timely and appropriate ICU care in high-risk surgical patients. This suggests that while the GTOS can be effectively utilized for all adult trauma patients, further improvements and verification across different demographics could enhance its predictive accuracy. Continued refinement and validation in diverse populations will be crucial in optimizing its utility and broadening its applicability.

Furthermore, the differences in the optimal GTOS cut-off values and their predictive performance metrics between this study and those of Arslan Erduhan et al. [16] and Egglestone et al. [42] can be attributed to several key factors. This study identified a GTOS cut-off of 104.5 for predicting overall mortality with high sensitivity (82.6%) and specificity (84.3%) in a broad cohort of trauma patients. The propensity analysis showed that patients with higher GTOS scores had significantly higher mortality rates and longer hospital stays, indicating worse outcomes even when baseline characteristics are similar. In contrast, Arslan Erduhan et al. [16] focused specifically on geriatric blunt trauma patients, reporting a lower GTOS cut-off of ≥95 for predicting 30-day mortality with a sensitivity of 76% and a specificity of 72.27%. Egglestone et al. [42] examined critically ill elderly trauma patients in ICUs, finding a much higher cut-off of ≥142, with a sensitivity of 74.2% and specificity of 54.9%. The differences may be due to variations in patient populations and settings, with this study including a broader trauma patient cohort, Arslan Erduhan et al. focusing on geriatric blunt trauma patients, and Egglestone et al. examining critically ill elderly patients in ICU settings. Future studies should refine the GTOS model to tailor it to different patient populations and clinical settings, establishing optimal cut-off values to enhance predictive accuracy and clinical utility.

Besides the GTOS and ISS, the outcomes of trauma patients are influenced by a complex interplay of factors, with pre-existing comorbidities playing a critical role. Our findings align with previous research that emphasizes the importance of considering these comorbidities in risk assessment and treatment planning. In our study, patients with conditions like HTN, DM, CHF, CAD, and ESRD showed higher odds of mortality in the univariate analysis, with ESRD remaining significant in the multivariate analysis. These results are consistent with Yang et al. [43], who demonstrated that cardiovascular comorbidities, including HTN and CHF, are associated with increased mortality rates and longer hospital stays, reflecting the additional physiological burden these conditions impose on trauma patients. Similarly, Elkbuli et al. [44] found that mortality rates among trauma patients increase with the number of comorbidities, particularly in those with three or more. Furthermore, trauma patients with poor long-term glycemic control face significantly higher risks of mortality and complications such as pneumonia, renal failure, urinary tract infections, and sepsis [45]. Spaetgens et al. [46] demonstrated that diabetes is independently associated with a 1.5-fold increased mortality risk in hip fracture patients, even after adjusting for comorbidities, BMI, and fracture type. Similarly, Merell et al. [47] found that poorly controlled diabetes (HbA1c > 8%) is linked to higher rates of complications and mortality. These studies collectively highlight the critical need to consider pre-existing comorbidities in the management and prognosis prediction of trauma patients.

This study has several limitations. First, the retrospective design may introduce selection bias and limit the ability to establish causality, as data accuracy and completeness depend on the quality of recorded information in the trauma registry. Second, the large discrepancy in the number of patients who survived against those who died may introduce bias, reducing this study’s statistical power and generalizability. Third, this study does not account for potential confounding factors such as vital signs, the mechanism of injury, variations in clinical practice, the existence of pre-hospital resuscitation, the execution of damage control surgery, the indication of transfusions, the presence of other comorbidities, or long-term rehabilitation outcomes. As a result, the GTOS, while effective in stratifying mortality risk, does not provide a comprehensive assessment of a patient’s overall condition or long-term prognosis beyond survival. Fourth, the study period spanning over two decades may include variations in medical practices and standards of care that could influence the outcomes, thereby affecting the consistency and reliability of the results. Furthermore, the design of the GTOS—age + ISS × 2.5 + 22 (if any PRBCs were transfused within 24 h of admission)—relies on data that may not be immediately available upon admission; therefore, the use of the GTOS is restricted in trauma patients with earlier mortality since the ISS specifically requires precise AIS coding and the inclusion of PRBC transfusion data is contingent upon a 24 h observation period. Additionally, the dataset was limited to in-hospital mortality, and we were unable to capture post-discharge deaths, which may result in an underestimation of the true mortality rate. Another limitation of this study is the variability in defining geriatric trauma. Geriatric patients represent a heterogeneous population with diverse injury patterns, comorbidities, and physiological responses. This highlights the need for further refinement to address specific subgroups within geriatric trauma populations. Future studies should also explore these comparisons to further evaluate the GTOS’s effectiveness and clarify its strengths and limitations compared to other predictive metrics. Finally, this study is based on a single trauma center, which limits the applicability of the results to different healthcare systems, underscoring the need for multi-center studies to validate and generalize the findings.

## 5. Conclusions

In conclusion, this study demonstrated that the GTOS is a simple effective tool for risk stratification in all trauma patients, allowing for faster allocation of attention and treatment. It is not limited to geriatric trauma patients but is equally effective in predicting mortality across a wide age range in adult patients with all trauma causes. However, its reliance on the ISS and transfusion data limits its applicability within the first 24 h following admission, making it maybe more suitable for early survivors rather than for guiding immediate clinical decisions during the initial resuscitation phase. Future research is needed to refine the GTOS for improved applicability in various kinds of patients or in different clinical settings.

## Figures and Tables

**Table 1:** Patient and injury characteristics of deceased and surviving patients.

Variables	Total *n* = 40,068	Death *n* = 818	Survival *n* = 39,250	OR (95% CI)	*p*
Sex					<0.001
Male, *n* (%)	21,761 (54.3)	545 (66.6)	21,216 (54.1)	1.70 (1.47–1.97)	
Female, *n* (%)	18,307 (45.7)	273 (33.4)	18,034 (45.9)	0.59 (0.51–0.68)	
Age, years (mean ± SD)	54.2 ± 19.4	63.4 ± 19.0	54.0 ± 19.4	-	<0.001
PRBC transfusion *, units (mean ± SD)	0.4 ± 2.0	4.0 ± 7.5	0.3 ± 1.6	-	<0.001
GTOS	77.2 ± 29.3	132.8 ± 29.9	76.1 ± 28.1	-	<0.001
Comorbidities					
CVA, *n* (%)	1620 (4.0)	45 (5.5)	1575 (4.0)	1.39 (1.03–1.89)	0.032
HTN, *n* (%)	12,125 (30.5)	332 (40.6)	11,883 (30.3)	1.57 (1.37–1.81)	<0.001
CAD, *n* (%)	1655 (4.1)	87 (10.6)	1568 (4.0)	2.86 (2.28–3.59)	<0.001
CHF, *n* (%)	271 (0.7)	14 (1.7)	257 (0.7)	2.64 (1.54–4.55)	<0.001
DM, *n* (%)	6678 (16.7)	180 (22.0)	6498 (16.6)	1.42 (1.20–1.68)	<0.001
ESRD, *n* (%)	800 (2.0)	67 (8.2)	733 (1.9)	4.69 (3.61–6.08)	<0.001
GCS, median (IQR)	15 (15–15)	6 (3–15)	15 (15–15)	-	<0.001
3–8, *n* (%)	1528 (3.8)	487 (59.5)	1041 (2.7)	54.00 (46.36–62.91)	
9–12, *n* (%)	1179 (2.9)	69 (8.4)	1110 (2.8)	3.17 (2.46–4.08)	
13–15, *n* (%)	37,361 (93.2)	262 (32.0)	37,099 (94.5)	0.03 (0.02–0.03)	
AIS ≥ 2					
Hand/Neck, *n* (%)	7326 (18.3)	632 (77.3)	6694 (17.1)	16.53 (14.00–19.50)	<0.001
Face, *n* (%)	3628 (9.1)	95 (11.6)	3533 (9.0)	1.33 (1.07–1.65)	0.010
Thorax, *n* (%)	4414 (11.0)	201 (24.6)	4213 (10.7)	2.71 (2.30–3.19)	<0.001
Abdomen, *n* (%)	2166 (5.4)	92 (11.2)	2074 (5.3)	2.27 (1.82–2.83)	<0.001
Extremity, *n* (%)	26,996 (67.4)	302 (36.9)	26,694 (68.0)	0.28 (0.24–0.32)	<0.001
ISS, median (IQR)	8 (4–9)	25 (16–29)	8 (4–9)	-	<0.001
1–15, *n* (%)	33,818 (84.4)	152 (18.6)	33,666 (85.8)	0.04 (0.03–0.05)	
16–24, *n* (%)	4436 (11.1)	162 (19.8)	4274 (10.9)	2.02 (1.70–2.41)	
≥25, *n* (%)	1814 (4.5)	504 (61.6)	1310 (3.3)	46.49 (39.96–54.08)	
Hospital stay (days)	8.8 ± 9.8	11.0 ± 15.8	8.7 ± 9.7	-	<0.001

AIS, Abbreviated Injury Scale; CAD, coronary artery disease; CHF, congestive heart failure; CVA, cerebrovascular accident; CI, confidence interval; DM, diabetes mellitus; ESRD, end-stage renal disease; GCS, Glasgow Coma Scale; GTOS, Geriatric Trauma Outcome Score; HTN, hypertension; IQR, interquartile range; ISS, Injury Severity Score; OR, odds ratio; PRBC, packed red blood cells; SD, standard deviation. * indicates the amount of PRBC transfusion within the first 24 h.

**Table 2:** Univariate and multivariate analysis of factors associated with mortality in trauma patients.

Mortality	Univariate Analysis			Multivariate Analysis		
	OR	95% CI	*p*	OR	95% CI	*p*
Male	1.70	(1.47–1.97)	<0.001	1.62	(1.37–1.92)	<0.001
Age	1.03	(1.02–1.03)	<0.001	1.01	(1.00–1.01)	0.209
PRBC transfusion *	1.25	(1.23–1.27)	<0.001	1.06	(1.04–1.08)	<0.001
GTOS	1.76	(1.71–1.80)	<0.001	1.37	(1.32–1.51)	<0.001
CVA	1.39	(1.03–1.89)	0.033	0.97	(0.69–1.36)	0.849
HTN	1.57	(1.37–1.81)	<0.001	0.97	(0.80–1.17)	0.747
CAD	2.86	(2.28–3.59)	<0.001	1.38	(1.05–1.81)	0.022
CHF	2.64	(1.54–4.55)	<0.001	1.73	(0.94–3.17)	0.077
DM	1.42	(1.20–1.68)	<0.001	1.00	(0.81–1.22)	0.973
ESRD	4.69	(3.61–6.08)	<0.001	4.47	(3.30–6.06)	<0.001
ISS	1.18	(1.17–1.19)	<0.001	1.09	(1.06–1.12)	<0.001

CAD, coronary artery disease; CHF, congestive heart failure; CVA, cerebrovascular accident; CI, confidence interval; DM, diabetes mellitus; ESRD, end-stage renal disease; GTOS, Geriatric Trauma Outcome Score; HTN, hypertension; ISS, Injury Severity Score; OR, odds ratio. * indicates the amount of PRBC transfusion within the first 24 h.

**Table 3:** Comparative analysis of the patients with a high and low Geriatric Trauma Outcome Score (GTOS) based on the optimal cut-off value of 104.5.

	GTOS			
	≥104.5 *n* = 7119	<104.5 *n* = 32,949	OR (95% CI)	*p*
Sex				<0.001
Male, *n* (%)	3627 (50.9)	18,134 (55.0)	0.85 (0.81–0.89)	
Female, *n* (%)	3492 (49.1)	14,815 (45.0)	1.18 (1.12–1.24)	
Age, years (mean ± SD)	72.1 ± 16.3	50.4 ± 17.8	-	<0.001
PRBC transfusion *, units (mean ± SD)	1.64 ± 4.1	0.1 ± 0.9	-	<0.001
Comorbidities				
CVA, *n* (%)	589 (8.4)	1022 (3.1)	2.87 (2.58–3.18)	<0.001
HTN, *n* (%)	3710 (52.1)	8505 (25.8)	3.13 (2.97–3.30)	<0.001
CAD, *n* (%)	659 (9.3)	996 (3.0)	3.27 (2.96–3.62)	<0.001
CHF, *n* (%)	105 (1.5)	166 (0.5)	2.96 (2.31–3.78)	<0.001
DM, *n* (%)	1853 (26.0)	4825 (14.6)	2.05 (1.93–2.18)	<0.001
ESRD, *n* (%)	253 (3.6)	547 (1.7)	2.18 (1.88–2.54)	<0.001
GCS, median (IQR)	15 (13–15)	15 (15–15)	-	<0.001
3–8, *n* (%)	1044 (14.7)	484 (1.5)	11.53 (10.31–12.88)	<0.001
9–12, *n* (%)	545 (7.7)	634 (1.9)	4.23 (3.76–4.75)	<0.001
13–15, *n* (%)	5530 (77.7)	31,831 (96.6)	0.12 (0.11–0.13)	<0.001
AIS ≥2				
Hand/Neck, *n* (%)	3689 (51.8)	3637 (11.0)	8.67 (8.18–9.18)	<0.001
Face, *n* (%)	768 (10.8)	2860 (8.7)	1.27 (1.17–1.38)	<0.001
Thorax, *n* (%)	1651 (23.2)	2763 (8.4)	3.30 (3.08–3.53)	<0.001
Abdomen, *n* (%)	721 (10.1)	1445 (4.4)	2.46 (2.24–2.70)	<0.001
Extremity, *n* (%)	4227 (59.4)	22,769 (69.1)	0.65 (0.62–0.69)	<0.001
ISS, median (IQR)	16 (9–24)	4 (4–9)	-	<0.001
1–15, *n* (%)	2936 (41.2)	30,882 (93.7)	0.05 (0.04–0.05)	<0.001
16–24, *n* (%)	2502 (35.1)	1934 (5.9)	8.69 (8.13–9.29)	<0.001
≥25, *n* (%)	1681 (23.6)	133 (0.4)	76.27 (63.78–91.21)	<0.001
Mortality, *n* (%)	681 (9.6)	137 (0.4)	25.33 (21.05–30.50)	<0.001
AOR of mortality ^†^	-	-	2.86 (2.20–3.73)	<0.001
Hospital stay (days)	14.0 ± 14.3	7.7 ± 8.1	-	<0.001

AOR, adjusted odds ratio; AIS, Abbreviated Injury Scale; CAD, coronary artery disease; CHF, congestive heart failure; CVA, cerebrovascular accident; CI, confidence interval; DM, diabetes mellitus; ESRD, end-stage renal disease; GCS, Glasgow Coma Scale; GTOS, Geriatric Trauma Outcome Score; HTN, hypertension; IQR, interquartile range; ISS, Injury Severity Score; OR, odds ratio; PRBC, packed red blood cells; SD, standard deviation; * indicates the amount of PRBC transfusion within the first 24 h. ^†^ mortality adjusted by sex, age, comorbidities, and GCS.

**Table 4:** Propensity score-matched analysis of patients based on the Geriatric Trauma Outcome Score (GTOS).

Propensity Score-Matched Cohort								
	GTOS							
	≥104.5 *n* = 3515		<104.5 *n* = 3515		OR (95% CI)		*p*	Standardized Difference
Male, *n* (%)	1888	(53.7)	1885	(53.6)	1.00	(0.91–1.10)	0.943	0.20%
Age, years	66.1	±15.9	66.0	±15.7	-		0.924	0.20%
CVA, *n* (%)	219	(6.2)	216	(6.1)	1.02	(0.84–1.23)	0.882	0.40%
HTN, *n* (%)	1604	(45.6)	1608	(45.7)	1.00	(0.91–1.09)	0.924	0.20%
CAD, *n* (%)	233	(6.6)	230	(6.5)	1.01	(0.84–1.22)	0.885	0.30%
CHF, *n* (%)	14	(0.4)	16	(0.5)	0.88	(0.43–1.79)	0.714	0.90%
DM, *n* (%)	884	(25.1)	887	(25.2)	1.00	(0.89–1.11)	0.934	0.20%
ESRD, *n* (%)	92	(2.6)	87	(2.5)	1.06	(0.79–1.43)	0.705	0.90%
GCS	15	(15–15)	15	(15–15)	-		0.875	0.40%
Outcomes:								
Mortality, *n* (%)	235	(6.7)	98	(2.8)	2.50	(1.96–3.18)	<0.001	-
Hospital stay (days)	14.8	±14.1	9.6	± 9.7	-		<0.001	-

CAD, coronary artery disease; CHF, congestive heart failure; CVA, cerebrovascular accident; CI, confidence interval; DM, diabetes mellitus; ESRD, end-stage renal disease; GCS, Glasgow Coma Scale; GTOS, Geriatric Trauma Outcome Score; HTN, hypertension; IQR, interquartile range; OR, odds ratio. Age is expressed as mean ± standard deviation; GCS is expressed as median with interquartile range.

## Data Availability

The de-identified data could be provided for academic research purposes via the corresponding author.
